# Rational Design of Toxoid Vaccine Candidates for *Staphylococcus aureus* Leukocidin AB (LukAB)

**DOI:** 10.3390/toxins11060339

**Published:** 2019-06-14

**Authors:** Shweta Kailasan, Thomas Kort, Ipsita Mukherjee, Grant C. Liao, Tulasikumari Kanipakala, Nils Williston, Nader Ganjbaksh, Arundhathi Venkatasubramaniam, Frederick W. Holtsberg, Hatice Karauzum, Rajan P. Adhikari, M. Javad Aman

**Affiliations:** Integrated Biotherapeutics Inc., Rockville, MD 20850, USA; skailasan@IntegratedBiotherapeutics.com (S.K.); Tom@IntegratedBiotherapeutics.com (T.K.); imukher@integratedbio.onmicrosoft.com (I.M.); gliao@IntegratedBiotherapeutics.com (G.C.L.); Tula@IntegratedBiotherapeutics.com (T.K.); nils.williston@gmail.com (N.W.); nader@IntegratedBiotherapeutics.com (N.G.); arundathi@IntegratedBiotherapeutics.com (A.V.); rick@IntegratedBiotherapeutics.com (F.W.H.); hkarauzum@IntegratedBiotherapeutics.com (H.K.)

**Keywords:** Leukocidin, *Staphylococcus aureus*, LukAB, LukGH, toxin neutralization, polyclonal antibody, toxoid vaccine

## Abstract

*Staphylococcus aureus* (SA) infections cause high mortality and morbidity in humans. Being central to its pathogenesis, *S. aureus* thwarts the host defense by secreting a myriad of virulence factors, including bicomponent, pore-forming leukotoxins. While all vaccine development efforts that aimed at achieving opsonophagocytic killing have failed, targeting virulence by toxoid vaccines represents a novel approach to preventing mortality and morbidity that are caused by SA. The recently discovered leukotoxin LukAB kills human phagocytes and monocytes and it is present in all known *S. aureus* clinical isolates. While using a structure-guided approach, we generated a library of mutations that targeted functional domains within the LukAB heterodimer to identify attenuated toxoids as potential vaccine candidates. The mutants were evaluated based on expression, solubility, yield, biophysical properties, cytotoxicity, and immunogenicity, and several fully attenuated LukAB toxoids that were capable of eliciting high neutralizing antibody titers were identified. Rabbit polyclonal antibodies against the lead toxoid candidate provided potent neutralization of LukAB. While the neutralization of LukAB alone was not sufficient to fully suppress leukotoxicity in supernatants of *S. aureus* USA300 isolates, a combination of antibodies against LukAB, α-toxin, and Panton-Valentine leukocidin completely neutralized the cytotoxicity of these strains. These data strongly support the inclusion of LukAB toxoids in a multivalent toxoid vaccine for the prevention of *S. aureus* disease.

## 1. Introduction

*Staphylococcus aureus*, which is a pervasive human pathogen, is a leading cause of life-threatening community and hospital-acquired infections world-wide. This Gram-positive bacterium is often associated with a range of diseases, from mild skin and soft tissue infections to invasive bacteremia, septic arthritis, endocarditis, and osteomyelitis [1]. Moreover, recent widespread emergence of multi-drug resistant strains, specifically methicillin and vancomycin-resistance, have not only complicated the use of available treatment options, but have also considerably raised the economic burden that is associated with staphylococcal infections [2,3,4]. Methicillin-resistant *S. aureus* (MRSA) causes ~80,000 invasive infections and 11,000 deaths per year in the United States alone [3].

*S. aureus* expresses a myriad of virulence factors, including cell surface attachment factors, capsular polysaccharides, enzymes, immune modulatory molecules, pore-forming toxins, and superantigens that aimed at establishing the infection or colonization as well as immune evasion [5]. Among the pore-forming toxins, *S. aureus* produces single component alpha hemolysin (Hla or α-toxin) and bicomponent pore-forming toxins (BCPFTs), Panton-Valentine leukocidin (PVL; composed of LukS-PV and LukF-PV), Leukocidin AB (LukAB), Leukocidin ED (LukED), and γ-hemolysins (HlgAB and HlgCB) [6]. While Hla is secreted as a monomer and oligomerizes on the plasma membrane of target cells upon interaction with its specific cellular receptor ADAM10 [7], the BCPFTs are produced from two distinct polypeptides, S (~32.4 kDa) and F (~34.6 kDa), which have β-barrel structures and hetero-oligomerize in a stepwise fashion with alternating S (LukS-PV, LukE, HlgA, HlgC, and LukA) and F (LukF-PV, LukD, HlgB, and LukB) subunits on the cell surface [8]. Following oligomerization, structural rearrangements within the C-terminal stem domain promote membrane insertion, resulting in ion efflux, disruption of the host cell lipid bilayer, and ultimately cell death.

Among the BCPFTs, LukAB is the most recently identified and it is one of the most potent members of the leukocidin family that kills human neutrophils, macrophages, monocytes, and dendritic cells [9,10]. While the majority of the different BCPFTs exhibit a high sequence identity of 70–80% among the S and F components, LukA and LukB share a low sequence identity of 30–40% with the other leukotoxins [6]. LukAB is also unique, in that it is secreted as a dimer in solution and it requires both S and F components for cell surface engagement in contrast to other BCPFTs [11]. The crystal structure of LukAB from USA300, which is a predominant methicillin-resistant *S. aureus* (MRSA) strain circulating in the United States (US), revealed an octameric arrangement of four LukAB dimers with two unique interfaces 1 (intra-protomeric) and 2 (inter-protomeric), which primarily govern the cap and rim domains [11]. Highly conserved residues among known LukAB variants that form salt bridges, a characteristic that is not found in other BCPFTs, hold these interfaces together. Of the two interfaces, residues within interface 1 were reported to be important for dimer formation, whilst interface 2 is important for octamerization. A surface-exposed residue E323 within the rim domain of LukA is critical to LukAB-mediated cytotoxicity by directly interacting with the integrin αM/β2 receptor (CD11b/CD18) on the surface of human neutrophils and monocytes (THP-1) [11,12,13,14]. In addition to the receptor binding properties, the rim domain harbors a high-affinity antibody epitope, which is also conserved among different LukAB sequence variants, and is suggestive of its role as an antigenic determinant as well as a site important for neutralization [15]. LukAB production not only allows the bacteria to escape from phagocytic killing by human neutrophils, but also lyse monocytes in a CD11b-targeted manner [9,12,13,16]. Furthermore, LukAB, along with α-toxin, were identified as key players in host macrophage dysfunction enabling USA 300 biofilm formation, highlighting yet another role of LukAB in circumventing the immune-mediated clearance in the host [17]. 

Highly virulent MRSA strains, like USA300, contribute to the clinical severity of SA infections by secreting a higher load of toxins to evade the innate immune system. Amongst other leukocidins, high titers to LukAB have been reported in acute and convalescent patients when compared to healthy controls [18]. Additionally, a study reported high levels of functional antibodies against LukAB in patients with invasive *S. aureus* infections as compared to healthy individuals or commercially available intravenous immunoglobulin (IVIG) [19]. Altogether, these studies suggest LukAB as a key protagonist in SA-related disease pathogenesis, bacterial survival, and persistence, and therefore a potential vaccine target. 

Here, iterative rounds of targeted single-amino acid and combination mutations within different functional domains of LukAB were designed in a structure-guided manner to identify the attenuated forms of LukAB. To this end, we first developed a novel production strategy that was based on co-expression from a single vector and a multistep purification process to generate tag-free, soluble dimers, and then functionally characterized them based on toxicity, thermostability, immunogenicity, and the ability to compete with cytotoxicity of wild type (WT) LukAB to identify several attenuated toxoids. Furthermore, we generated a polyclonal antibody against a selected attenuated LukAB mutant that demonstrated neutralizing activity towards supernatants from several laboratory and clinical strains, including USA300, which is responsible for the current CA-MRSA outbreak in the United States. Our findings further indicate that a multivalent toxoid vaccine targeting all the pore-forming toxins is needed to fully neutralize the cytotoxicity of different clinical strains of *S. aureus* toward neutrophils and monocytes.

## 2. Results

### 2.1. Expression of LukAB using pET Duet versus pET24a(+) Dual Plasmid System

Unlike other staphylococcal leukotoxins, the LukAB proteins, when expressed as separate subunits, do not fold properly, and expression is directed to inclusion bodies [12]. In the past, we also noted that, when these proteins are refolded together from solubilized inclusion bodies, the resulting yield and toxicity of the product is lower than that of the co-expressed [12] and co-purified LukAB proteins [20]. Therefore, we compared purified LukAB proteins from two expression vectors, pET-Duet expressing the subunit from the same plasmid, and pET24a (+) dual plasmid expression systems and observed reasonably good and equivalent yields of LukAB_WT_ proteins (Table S1). Both of the systems have their merits. With pET-Duet, as both subunits are expressed from a single plasmid while using the same antibiotic marker, and therefore easier to scale up from a manufacturing prospective. On the other hand, with pET24a (+) dual system, as LukA and LukB are co-expressed within the same cell by two different plasmids, it is a better system for introduction of mutations while screening a mutation library. Additionally, we found that the toxicity and physicochemical properties of LukAB_WT_ from the two systems are comparable (Table S1). Hence, we used a pET24a (+) dual system during mutant screening and pET-Duet for production.

### 2.2. Rational Design of Attenuated LukAB Toxoids

**Figure 1:**
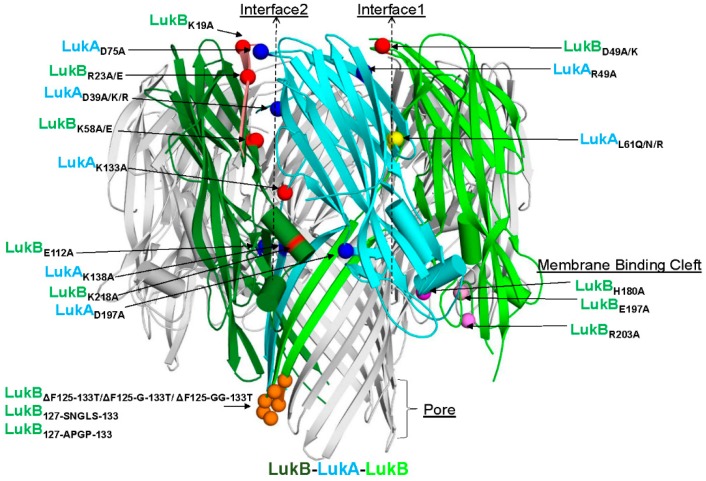
The repertoire of selected Leukocidin AB (LukAB) residues for mutagenesis. Cartoon representation of the crystal structure of LukAB (PDB: 4TWI) with polypeptides LukA and LukB shown in blue and green, respectively, along with the neighboring LukB polypeptide within the octameric ring shown in dark green. The α-carbon atoms of residues selected for mutation are labeled and depicted as spheres. Of the selected mutations, basic residues are shown in red, acidic in blue, hydrophobic in yellow, surface-loop residues near the pore in orange and those part of the membrane binding cleft in pink. As the complete LukB N terminal sequence was not entirely resolved in this crystal structure, mutated residue LukB_K12_ is not shown in this depiction.

**Figure 2:**
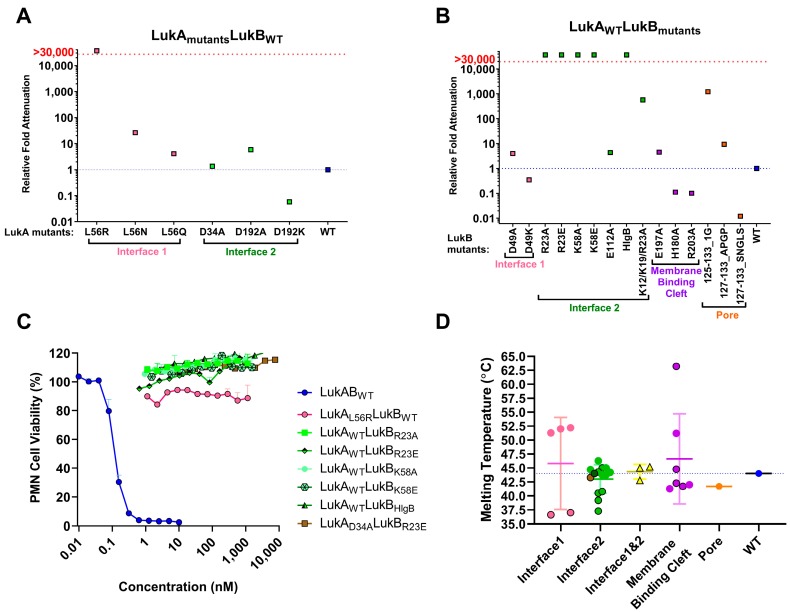
Characterization of LukAB mutants. Polymononuclear neutrophils (PMNs) were treated with increasing concentrations of LukAB_WT_ or mutants within the concentration range of 1000 to 0.001 nM to calculate toxicity EC_50_s. Results represent the fold attenuation (mutant EC_50_/WT EC_50_) and shown in (**A**) for mutants of LukA combined with wild type (WT) LukB and in (**B**) for mutants of LukB combined with WT LukA mean. Calculated EC50 values are listed in Table 1 and were measured in 3 independent experiments. (**C**) Toxicity profiles of LukAB_WT_ or the most highly attenuated mutant proteins at the indicated concentration range were tested in PMNs as % cell viability. (**D**) Thermostability measurements using differential scanning fluorimetry (DSF) to calculate melting temperatures (T_m_s) are plotted for the different, individual LukAB mutants within the different domains. Average values and spread of recorded Tms indicated for each domain. For Interface 1, outlined circles indicate non-LukA_L61_ mutants. For interface 2, mutants LukA_D39A_LukB_R23E_ and LukA_wt_LukB_HlgB_ have been shown as outlined circles in brown and dark green, respectively.

**Figure 3:**
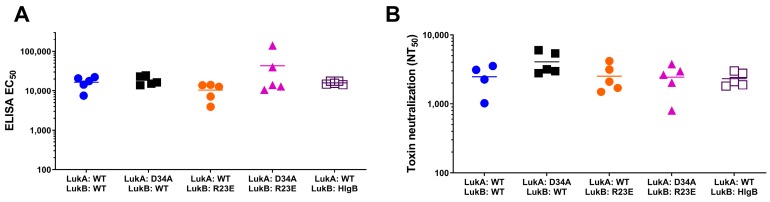
Immunogenicity titers for LukAB mutants. (**A**) Enzyme-Linked Immunosorbent Assay (ELISA) and (**B**) toxin neutralization assays (TNA) titers calculated for select mutants with titers for each individual mouse per group and average titers represented by the appropriate symbol and horizontal line, respectively. The Y axis is shown in log scale.

**Figure 4:**
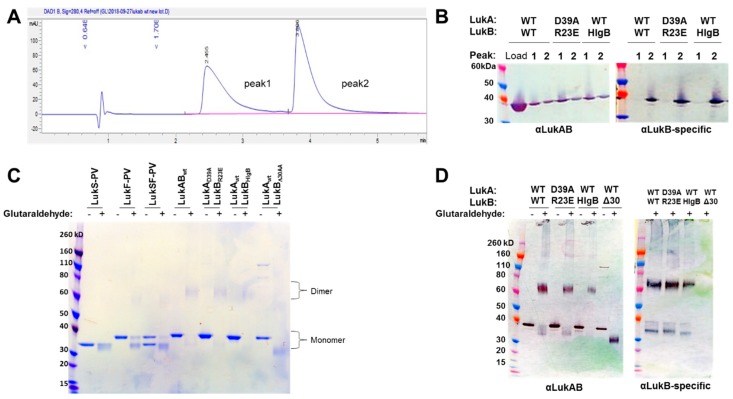
Biophysical characterization of selected LukAB mutants. (**A**) Example of the reverse-phase HPLC chromatograph of LukAB_WT_ highlighting the two fractionated peaks, 1 and 2. (**B**) Immunoblots treated with either 1 µg/mL of αLukAB or 0.1 µg/mL of αLukB-specific pAB highlighting peak 1 and 2 fractionated samples of LukAB_WT_, LukA_D39A_LukB_R23E_, and LukA_wt_LuK_HlgB_. Molecular weights are also indicated. Individual proteins as indicated were incubated with or without 0.25% glutaraldehyde and analyzed by (**C**) SDS-PAGE without boiling and (**D**) Western blot using αLukAB or 0.1 µg/mL of αLukB-specific pAB.

The objective of this study was to generate attenuated forms of LukAB as the vaccine candidates. To this end, we used the available crystal structure of LukAB heterodimer (PDB: 5K59) and octamer (PDB: 4TW1, Figure 1) as the structural framework to design mutations that could potentially reduce the cytotoxicity, while maintaining structural integrity and other indispensable functional characteristics, such as dimer formation, receptor binding, and immunogenicity. The mutations were predominantly made within four domains of the LukAB dimer: interface 1 (protomer-protomer interface), interface 2 (dimer-dimer interface), membrane binding cleft that interacts with the lipid bilayer, and the stem domain, which forms a β-barrel pore inserted into the lipid bilayer (Figure 1). All of the 39 mutants designed, including combinations, target residues that are highly conserved among more than 40 LukAB_WT_ sequences from different *S. aureus* strains for which annotated genomic data are available (Figure S1). Additionally, the majority of these mutants were expressed as soluble proteins and they could be purified at reasonable yield, unless otherwise indicated (Table 1). All of the mutants were analyzed for (1) purity measured by SDS-PAGE (data not shown) and yield (Table 1), (2) toxicity levels tested using a cytotoxicity assay in induced HL-60 cells differentiated into polymononuclear neutrophils (PMNs), as described in the Methods (Figure 2A–C and Table 1), (3) thermostability measured by differential scanning fluorimetry (DSF) (Figure 2D), and (4) immunogenicity levels in mice (Figure 3 and Table 1). The non-toxic LukAB vaccine candidates formulated in Alhydrogel® (Al(OH_3_)) as an adjuvant were examined for the induction of neutralizing antibodies. Groups of five female ICR (CD-1) mice were intramuscularly immunized (IM) with 10µg of LukAB mutants or LukAB_WT_ in 50µg of Al(OH)_3_ three times at two-week intervals and sera was collected 10 days after final immunization. Antibody-binding and -neutralizing titers were measured either for individual mice or from pooled sera for each mutant group by ELISA and toxin neutralization assays (TNA), respectively, as described in the Methods.

#### 2.2.1. Mutations within Interface 1 of LukAB 

We targeted L61 on Interface 1, a key residue that aligns with H35 of alpha hemolysin (α-toxin; Hla) and T28 of Panton-Valentine leukocidin (PVL) S subunit (LukS-PV) [21]. N terminal residues H35 and H48 of Hla are imperative to protomer-protomer interactions and their substitution results in the loss of hemolytic activity due to the formation of incomplete, larger heptameric rings, as observed by electron microscopy [22]. The substitution of T28 of LukS-PV with a bulky residue, such as phenylalanine, is also known to affect toxicity due to steric or electrostatic repulsions that might interfere with oligomerization [21,23]. We designed three substitutions—L61N, L61Q, and L61R—to incrementally disrupt the hydrophobic pocket found within LukAB interface 1 (Figure 1). The second mutagenesis site targeted within interface 1 was the salt bridge comprised of LukA_R49_ and LukB_D49_ (Figure 1). We disrupted the electrostatic interactions by alanine and lysine substitutions at LukB_D49_. Of these mutants, LukA_L61Q/N_LukB_wt_, and LukA_wt_LukB_D49A/K_, exhibited similar or enhanced cytotoxicity, respectively, in PMNs with EC_50_s in the range of 0.01–0.7 nM as compared to WT (EC_50_ = 0.027 nM) (Table 1, Figure 2A,B). In contrast, the introduction of a bulky, positively charged residue, like arginine (LukA_L61R_LukB_wt_), reduced the toxicity by >30,000-fold as compared to WT (Table 1, Figure 2A). Additionally, thermostability measurements by DSF indicated that the mutation of L61 of LukA increases protein stability, because mutants LukA_L61R/N/Q_LukB_wt_ recorded melting temperatures (Tm) that were in the range of 51.3–52.2 °C, representing a positive thermal shift of 7.3–8 °C as compared to LukAB_WT_ (Tm = 44 °C) (Figure 2D). However, when tested for immunogenicity in mice, the highly attenuated mutant LukA_L61R_LukB_wt_ resulted in ~50% reduction of total IgG and TNA titers as compared to LukAB_WT_ (Table 1). Therefore, this mutant was not further considered as a vaccine candidate.

#### 2.2.2. Pore and Membrane Binding Domain LukAB Mutants

It has been reported that the membrane binding cleft in the rim domain of both Hla and LukF-PV binds to polar head groups on the lipid bilayer [23]. Hence, we targeted three LukB residues within the membrane binding cleft, namely, H180A, E197A, and R203A, with the anticipation of reducing toxicity as membrane lipid binding is a preliminary step in pore formation (Figure 1). When combined with LukA_WT_, the LukB_E197A_ mutation modestly attenuated toxicity (~4.5-fold), while the other two mutations failed to attenuate cytolytic activity in PMNs with EC_50_s in the range of LukAB_wt_ (Figure 2B; Table 1). The mutations were also designed within a surface loop on LukB between residues 125-133 (FSINRGGLT) in the β-barrel pore in order to obturate the cytoplasmic edge of the pore (Figure 1, Figure S1). To this end, we made deletion mutants LukB_Δ125-133_, LukB_Δ125-G-133_, and LukB_Δ125-GG-133_, supplemented with or without additional glycine(s) to introduce flexibility between residues D124 and G134 and to maintain structure stability. Of these mutants, only LukA_wt_LukB_Δ125-G-133_ could be expressed, although poorly, and demonstrated moderate 1218-fold attenuation with TNA titers similar to wild type (Figure 2B, Table 1). This mutant was discarded due to expression and purification difficulties. We also substituted residues 125–133 with a short Type-1 β-turn sequence (APGP) as LukA_wt_LukB_127-APGP-133_ or with the analogous loop sequence of HlgB (SNGLS) as LukA_wt_LukB_127-SNGLS-133_. While LukA_wt_LukB_127-APGP-133_ diminished the cytotoxicity by ~10-fold, the EC_50_ for LukA_wt_LukB_127-SNGLS-133_ toxicity was enhanced by ~100 fold (Figure 2B). Overall, the mutagenesis of the targeted residues within the membrane binding domain and pore region failed to provide viable vaccine candidates for either a lack of proper attenuation or poor yield.

#### 2.2.3. Mutations within Interface 2 of LukAB

We made several mutations on LukB to target the LukAB dimer-dimer interface and to potentially disrupt its ability to oligomerize on the cell surface (Figure 1). First, we targeted three salt bridges that were observed in the crystal structure on interface 2, namely, LukA_D75_-LukB_R23_ and LukA_D39_-LukB_K58_, which lie within the cap domain and LukA_K133_-LukB_E112_ that lies closer to the rim domain (Figure 1). Mutations that were made to disrupt the latter salt bridge, LukA_wt_LukB_E112A_, failed to significantly attenuate toxicity (Table 1, Figure 2B), and we therefore focused our efforts on those on the apical side (cap). The disruption of electrostatic interactions at position R23 in the form of mutant LukA_wt_LukB_R23A_ or the reversal of positive charge with mutant LukA_wt_B_R23E_ resulted in an EC_50_ greater than 1000 nM representing >30,000-fold attenuation of cytotoxicity in PMNs (Table 1; Figure 2B). Visual inspection of the crystal structure (PDB 5K59) highlighted two additional positively charged residues in the vicinity of LukB_R23_, K12, and K19, which may also contribute to a positively charged surface juxtaposing LukA_D75_. K12 and K19 line the inner face of the β-barrel core. Therefore, we generated a triple mutation at the N terminus of LukB, LukA_wt_LukB_K12A/K19A/R23A_ to disrupt this basic amino acid patch. However, it resulted in an EC_50_ of 15.6 nM with a moderate attenuation of 573-fold as compared to LukAB_wt_ (Table 1, Figure 2B). Altogether, these data indicated that LukB_R23_ at the N terminus plays the most critical role in maintaining the ionic interactions at the cap domain of LukB and it is central to its cytotoxicity, but a more disruptive mutation, like R23E, is required for optimal attenuation (Table 1). However, a larger area of disrupted positive charges does not augment the level of cytotoxic attenuation. Moreover, relative antibody and neutralizing titers for LukA_wt_LukB_K12A/K19A/R23A_ and LukA_wt_LukB_R23A_ were considerably lower than LukA_wt_LukB_R23E_ and LukAB_wt_ (Table 1), and therefore these mutants were no longer pursued as potential vaccine candidates. The average immunogenicity titers for LukB_R23E_-containing mutants were comparable to WT (Figure 3).

Similarly, we generated LukA_wt_LukB_K58A_ for salt bridge LukA_D39_-LukB_K58_, which exhibited a >30,000-fold reduction of toxicity, suggesting a critical functional role for K58 (Figure 2B; Table 1). Similar results were obtained with glutamic acid substitution at K58, indicating the importance of a positive charge at this position (Figure 2B; Table 1). Protein stability and IgG titers were found to be analogous to WT, while neutralizing titers for LukA_wt_LukB_K58A_ were ~2.61-fold better than LukAB_wt_ (Table 1). However, the overall protein yields that were obtained for LukA_wt_LukB_K58A/E_ were consistently five-times lower than LukAB_wt_ and therefore not pursued (Table 1). Surprisingly, despite the indispensable role of K58, the reciprocal mutant LukA_D39A_LukB_wt_ showed toxicity levels that were comparable to WT with an EC_50_ of 0.037 nM (Table 1). The IgG titers that were elicited by LukA_D39A_LukB_wt_ were as high as LukAB_WT_, but the neutralizing titers were higher at 1.4-fold better as compared to LukAB_wt_ (Figure 3, Table 1). By combining LukA_D39A_ with attenuating LukB_R23E_ mutation within the same interface, we observed an enhancement of the overall neutralizing titers of LukA_wt_LukB_R23E_ by a factor of 2, while retaining >30,000-fold attenuation in toxicity (Table 1, Figure 2C). Altogether, our observations suggest that disruption of the positively charged residues on LukB are more effective for designing LukAB toxoids than the corresponding residues on LukA. 

When considering our observations, where the LukB N terminal residues, K12, K19, and R23 reduced toxicity, we wanted to examine whether the LukB N terminus is directly involved in mediating cytotoxicity. Both available crystals structures of LukAB are missing the first 10–15 residues of the LukB N terminus, which suggests that this region may be highly unstructured or disordered in the crystal form and it may be stabilized upon binding or oligomerization. Moreover, the superposition of the crystal structures of LukB (4TWI) and HlgB suggested high structural similarity, except for the loops at the rim domain. Taking these observations together, we further investigated the role of the first 30 N terminal residues of LukB by either deleting them (LukA_wt_LukB_Δ30AA_) or replacing with the analogous sequence of HlgB (LukA_wt_LukB_HlgB_) (Figure S1). HlgB has ~70% sequence divergence from LukAB in this region and it does not contain the analogous bulky, positively-charged residues at the same location as LukB. While we were unable to purify a meaningful amount of LukA_wt_LukB_Δ30aa_ due to the poor expression of LukB_Δ30aa_ (data not shown), mutant LukA_wt_LukB_HlgB_ was well expressed and it exhibited a cytotoxicity EC_50_ of >1000 nM, representing >30,000-fold attenuation (Figure 2B, Table 1). The protein stability (Figure 2D) and relative immunogenicity levels were also comparable to LukAB_WT_ (Figure 3, Table 1). We also tested whether the N terminal HlgB sequence mediates any HlgB specific neutralizing responses; however, no such response was observed, which suggested a lack of neutralizing epitopes within this region of HlgB.

#### 2.2.4. Fully Attenuated Interface 1&2 Combination Mutants

We tested the cytotoxicity of several attenuated mutations on HL-60-derived PMNs at the highest concentration possible and detected no residual toxicity for mutants LukA_wt_LukB_R23A/E,_ LukA_wt_LukB_K58/E_, LukA_D39A_LukB_R23E_, and LukA_wt_LukB_HlgB_. Mutant LukA_L61R_LukB_wt_ retained 6–4% toxicity at very high concentrations (Figure 2C). Altogether, based on the data from expression, toxicity, stability, and immunogenicity experiments, we concluded that mutants LukA_wt_LukB_R23E_, LukA_D39A_LukB_R23E_, and LukA_wt_LukB_HlgB_ show the most significant attenuation in PMN cytotoxicity, and LukA_D39A_LukB_R23E_ and LukA_wt_LukB_HlgB_ also retain the ability to produce high-titer, neutralizing antibodies in ICR mice. Therefore, we focused our efforts to further characterize these two mutants.

### 2.3. Biophysical Characterization of Selected LukAB Vaccine Candidates

Next, we wanted to confirm whether the selected mutations within the attenuated LukAB candidates affect protein dimerization in solution. This is important, as the elicitation of effective neutralizing antibodies is dependent on maintaining the heterodimer structure [15]. Initial efforts to detect the LukAB dimer by size exclusion chromatography were unsuccessful, as LukAB could not be resolved in SEC-HPLC while using the hydrophilic polymer-coated AdvanceBio SEC column (PL1180-5301) or silica-based Agilent ProSEC300 (PL1147-6501). Additionally, no peaks were detected on the A_280_ chromatograph when increasing the salt conditions (up to 500mM NaCl) and/or varying pH 6–7.0 were implemented. Therefore, we sought to fractionate the proteins by reverse-phase (RP) HPLC. We first fractionated LukAB_WT_, LukA_D39A_LukB_R23E_, and LukA_wt_LukB_HlgB_ while using acetonitrile that resolved into two prominent peaks (Figure 4A). We analyzed the two RP-HPLC fractions by Western blot while using two isolated rabbit polyclonal antibodies—one that cross-reacts to LukA and LukB (referred to as αLukAB) and another LukB-specific (referred to as αLukB-specific) antibody generated, as described in the Methods. As seen in Figure 4B, the results suggested that the first and second peak consist of LukA and LukB, respectively, and the mutants are comparable to WT. Once we confirmed that our expressed form of LukAB is composed of both components, we wanted to verify its oligomerization level in solution. Wild-type and mutant LukAB proteins were incubated with glutaraldehyde, as described in the Methods section to cross-link the LukA and LukB components. LukS-PV and LukF-PV were used as the controls, as they are known to exist as monomers in solution and only dimerize in the presence of the host cellular receptor. As shown in Figure 4C,D, the examination of the glutaraldehyde cross-linked products on SDS-PAGE and Western blot indicated that LukAB_WT_ exists as a dimer (~77 kDa). In contrast, LukS-PV (MW = 70.6 KDa) and LukF-PV (MW = 73.9 KDa) remained largely monomeric in the presence of glutaraldehyde. Mutants LukA_D39A_LukB_R23E_ and LukA_WT_LukB_HlgB_ are similar to LukAB_WT_ as dimers, indicating that they retain the heterodimeric structure that is important for eliciting neutralizing antibodies. Additionally, we also ran the poorly expressed LukA_WT_LukB_Δ30aa_ mutant, in which the LukA component fails to associate with LukB_Δ30AA_, suggesting a previously unknown role for the N terminus of LukB in protomer-protomer interaction (Figure 4C,D).

### 2.4. Functional Characterization of Selected LukAB Vaccine Candidates

**Figure 5:**
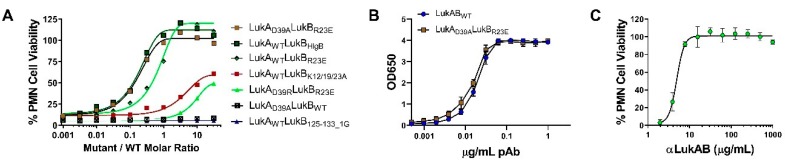
Functional characterization of selected LukAB mutants in PMNs. (**A**) LukAB_WT_ and mutant competition binding to PMNs. (**B**) ELISA and (**C**) TNA results testing the quality of binding and neutralization of αLukAB polyclonal antibodies to LukAB_WT_ and LukA_D39A_LukB_R23E_. All the error bars represent measurements made in duplicates.

The ability of mutants to bind to the cellular receptor CD11b would indicate an intact receptor binding site, which could be important for the induction of neutralizing antibodies. Therefore, we sought to determine whether the selected mutants compete with LukAB_WT_ for receptor binding. Direct competition assay between the mutant and WT, followed by the detection of bound protein by flow cytometry is not possible because of the rapid cytotoxic effect of LukAB. We reasoned that competition between WT and mutant for receptor binding should result in the reversal of LukAB toxicity when the toxicity is measured in the presence of excess amount of mutant. To this end, various concentrations (0.4 pM-13 nM) of the mutants LukA_D39A_LukB_R23E,_ LukA_wt_LukB_HlgB,_ LukA_wt_LukB_R23E_, LukA_wt_LukB_125-133_1G_, LukA_D39R_LukB_R23E_, and LukA_wt_LukB_K12/19/R23A_, which are fully attenuated, or LukA_D39A_LukB_wt_, which retains toxicity, were incubated with PMNs along with a fixed concentration of 0.42 nM LukAB_WT_. Relative toxicity of LukAB in the presence of various mutants was then plotted against the molar ratio of mutant to wild type (Figure 5A). Consistent with our hypothesis, the mutants suppressed LukAB toxicity in a dose dependent manner. Of these mutants, LukA_D39A_LukB_R23E_ and LukA_wt_LukB_HlgB_, followed by LukA_wt_LukB_R23E_, exhibited the most dramatic effect, with 50% reversal of LukAB_WT_ toxicity at a mutant/WT ratio of 0.17, 0.18, and 0.64, respectively. Even the full inhibition of LukAB toxicity was observed with the first two mutants at molar ratios below 1. These data suggested that, at least for these three mutants, the reversal of toxicity cannot be entirely explained by the competition for receptor binding. A potential explanation for these data is that these mutants may be able to form mixed, defective oligomeric structures on the cell surface with wild type LukAB dimers. Mutants LukA_D39R_LukB_R23E_ and LukA_wt_LukB_K12/19/R23A_ were less efficient in competing with WT with 50% reversal only being observed at the highest mutant/WT molar ratio of >30 (high mutant excess). On the other hand, attenuated LukA_WT_LukB_125-133_1G_, having a deletion in the loop that is involved in pore formation, failed to reverse the lytic effect of LukAB, even at high concentrations, which suggested that this mutation can neither compete with receptor binding nor form mixed defective oligomers. As expected, LukA_D39A_LukB_wt_, a mutant that retains substantial toxicity, had no reverting effect on LukAB activity.

Out of the two best attenuated candidates, LukA_D39A_LukB_R23E_ and LukA_wt_LukB_HlgB_, we tested the ability of LukA_D39A_LukB_R23E_ for its ability to generate neutralizing antibodies in rabbits. The total IgG was purified from the sera of rabbits immunized with the LukAB mutant showing high and equal binding to both wild type and mutant LukA_D39A_LukB_R23E_ (Figure 5B), in addition to being able to effectively neutralize LukAB_WT_ at an NT_50_ of 4.9 µg/mL (Figure 5C).

**Figure 6:**
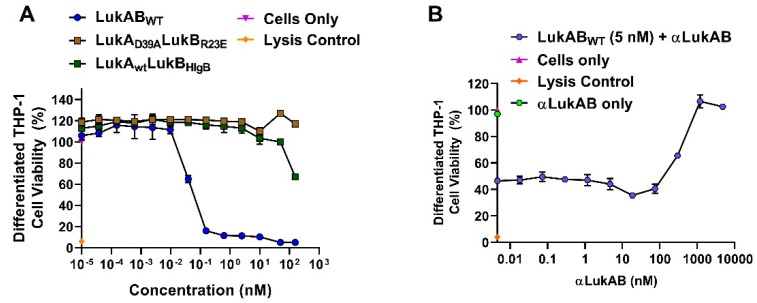
LukAB-mediated cytotoxicity and TNA in THP-1. (**A**) Differentiated THP-1 cells intoxicated with purified LukAB_WT_, LukA_D39A_LukB_R23E_, or LukALukB_HlgB_ proteins in a dose-dependent manner to measure cell viability and cytotoxicity. (**B**) Cells intoxicated with a constant concentration of LukAB_WT_ (5 nM) in the presence of titrated αLukAB polyclonal antibody starting at a concentration of 5000 nM and diluted 2-fold. For all graphs, percent cell viability is plotted as a function of log concentration shown in nM and error bars represent measurements made in duplicates. Readout using CTG luminescence. Lysis control shown contains cells incubated with 0.2% TritonX-100.

Published work using SA Newman strain or individual isogenic mutants that were deficient of LukAB, ⍺-toxin, HlgABC, or LukED demonstrated that SA directly kills human derived monocytes by promoting necrotic cell death in primary CD14^+^ human monocytes in a LukAB-CD11b-dependent manner [14]. Therefore, we wanted test whether our best mutants are also attenuated in the THP-1 cells. To test this, while using flow cytometry, we first confirmed that CD11b expression increased from ~13% to 85% upon the differentiation of THP-1 cells using PMA, which results in the macrophage-like phenotype, mimicking primary human macrophages (Figure S2). Consistently, the measured CD14^+^ levels were also high for these cells. Cytotoxicity assay with purified LukAB and mutants, LukA_D39A_LukB_R23E_ and LukA_wt_LukB_HlgB_, showed an EC_50_ of 0.04 nM for LukAB and the complete loss of toxicity for LukA_D39A_LukB_R23E_ at concentrations as high as 160 nM (Figure 6A). LukA_WT_LukB_HlgB_ exhibited ~33% residual toxicity at the highest concentration tested. Similar to HL-60 cells, polyclonal antibodies that were generated against LukA_D39A_LukB_R23E_ were able to neutralize LukAB_WT_-mediated toxicity in differentiated THP-1 cells (Figure 6B). Together, these data show that LukA_D39A_LukB_R23E_, as a vaccine candidate, can generate potent, neutralizing antibodies that are capable of reversing LukAB_WT_-mediated toxicity and preventing monocyte lysis.

### 2.5. Attenuated LukAB Candidate as a Component of a Multivalent Toxoid Vaccine

**Figure 7:**
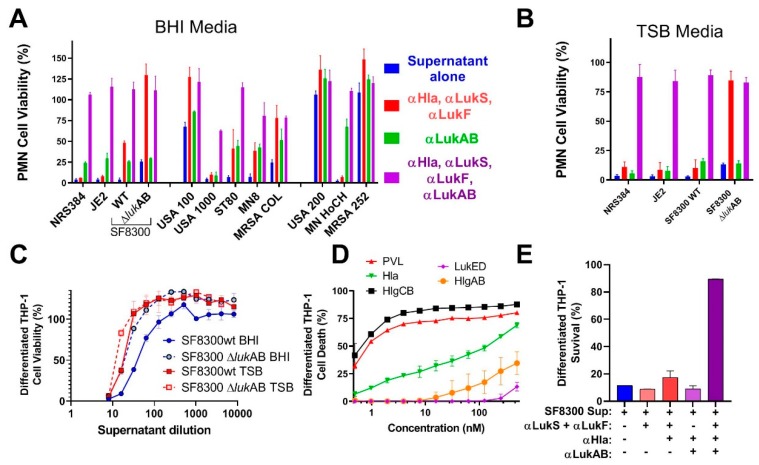
Toxicity and TNA of bacterial culture supernatants. Bacterial culture supernatants grown in (**A**) BHI or (**B**) TSB media incubated with indicated polyclonal antibodies to measure relative cytotoxicity and neutralization post a 3 h incubation in PMN cells. Error bars indicate measurements made in triplicates. (**C**) Toxicity profiles of SF8300 wild-type and LukAB-deficient isogenic mutant grown in BHI or TSB media on differentiated THP-1 cells measured 4 h post incubation. Error bars represent measurements made in triplicates. (**D**) Measurement of toxicity of purified toxoids such as α-toxin and BCPFTs on differentiated THP-1 cells. CTG readout was carried out 1 h post incubation with toxoids and error bars indicate measurements made in duplicates. (**E**) TNA results of supernatants from SF8300WT or SF8300ΔlukAB strains grown in TSB and BHI media and incubated with αLukAB, αLukS, αLukF, or αHla, or a combination as labeled for 4 h to measure extent of neutralization indicated by cell viability measurements. Error bars represent measurements made in duplicates.

Multiple pore-forming toxins, including Hla, PVL, LukED, HlgAB, HlgCB, and LukAB, mediate the cytotoxic activity of SA. While LukAB and PVL are most lytic to PMNs, this activity is shared by several other leukotoxins [6]. We measured the ability of antibodies against LukAB and other leukotoxins to neutralize leukotoxicity in HL-60 and THP-1 cells induced by bacterial culture supernatants from different standard clinical SA strains, as listed in Table S2, to establish the potential contribution of a LukAB toxoid to a multivalent toxoid vaccine. Bacterial supernatants were produced by growing the cells to stationary phase in BHI or TSB media, sterile-filtered, and used in PMN cytotoxicity assay in the presence of rabbit polyclonal antibodies raised against LukA_D39A_LukB_R23E_ (αLukAB), two PVL subunit toxoids LukS_mut9_ (αLukS) and LukF_mut1_ (αLukF), which we previously reported [21], or the toxoid Hla_H35LH48L_ (αHla). Besides three isolates of USA300, we also used a LukAB isogenic knock out of the USA300 strain SF8300 (SF8300ΔlukAB). As shown in Figure 7A, supernatants from these three isolates, as well as SF8300ΔlukAB, were highly toxic toward PMNs. Either a combination of antibodies against PVL and α-toxin or LukAB alone had modest neutralizing activity towards supernatants of the three wild-type strains, while a combination of the antibodies against all of these toxins was able to fully neutralize the cytotoxicity (Figure 7A). In contrast, when the lukAB gene was deleted, the cytotoxicity was entirely neutralized by antibodies to PVL and Hla. These results were also confirmed using TSB media for bacterial growth (Figure 7B). These data indicated that efficient neutralization of leukotoxicity of USA300 requires a broadly neutralizing antibody response, including LukAB. In contrast to USA300, LukAB did not appear to significantly contribute to leukotoxicity mediated by USA100, ST80, USA1000, and COL strains (Figure 7A). Total cytotoxicity induced by USA200, MNHoCH, and MRSA252 was too low to determine the role of LukAB. 

We next tested whether αLukAB also protects differentiated THP-1 cells against cytotoxic activity in the bacterial culture supernatants. For this, we examined the cell viability in presence of supernatants from SF8300 and SF8300ΔlukAB grown in TSB or BHI media to gauge the level of LukAB-mediated toxicity in differentiated THP-1 cells. The cytotoxicity of WT strain was 2.6-fold or 1.8-fold fold higher than SF8300ΔlukAB when grown in BHI or TSB, respectively (Figure 7C), which suggested that LukAB, despite its potent cytotoxicity towards THP-1 cells (Figure 6A), only partially contributes to monocyte killing by SF8300 culture supernatant and other pore-forming toxins may also contribute to overall cytotoxicity. To this end, we evaluated the cytotoxicity of other pore-forming toxins Hla, PVL, HlgAB, HlgCB, and LukED. As shown in Figure 7D, of these toxins, HlgCB and PVL, followed by Hla, displayed the strongest cytotoxicity toward differentiated THP-1 cells, while HlgAB and LukED showed poor activity. However, all of these toxins were far less potent than LukAB (EC_50_ 0.04 nM, Figure 6A). We next evaluated the ability of antibodies against PVL, Hla, and LukAB to neutralize the toxicity of SF8300 supernatant toward THP-1 cells. At a 1:10 dilution of the supernatant that caused >90% cytotoxicity in THP-1 cells, only a combination of all these antibodies was able to fully neutralize the toxic activity of the supernatant (Figure 7E). These data, again, point to the importance of neutralizing multiple pore-forming toxins for the complete protection of monocytic cells.

## 3. Discussion

The evolution and emergence of MRSA strains has become a major challenge to global health. Among the diverse virulence factors that were secreted by SA to subvert the encountering host defense system, α-toxin/Hla and the bicomponent leukotoxins, such as LukAB, HlgAB, HlgCB, LukED, and PVL, are the most potent, because they specifically target and kill innate immune cells and disrupt biological barriers by lysing epithelial and endothelial cells [24], as well as keratinocytes [25]. Prior efforts toward vaccine development for *S. aureus* have myopically focused on promoting the opsonophagocytic uptake of the bacteria and the subsequent killing by phagocytic cells, an approach that has been successful for several pathogens, such as *S. pneumoniae*, *N. meningitidis*, and *H. influenzae* B. However, these efforts have failed to deliver an effective vaccine for *S. aureus* and at least one of the experimental vaccines, Merck V710, led to increased mortality in vaccinated individuals who developed SA infection [26], which suggested possible immunopathology. Several epidemiological studies indicate that the SA toxins are important vaccine targets [27,28,29]. We have previously developed vaccine candidates for PVL subunits that elicit cross-reactivity to HlgAB, HlgCB, and LukED [21]. LukAB plays a significant role in mediating SA virulence and its potency is most comparable to PVL [8]. Unlike PVL, which is carried by phages and is only found in 5–15% clinical isolates, LukAB is chromosomally encoded and found in the majority of the isolates [6]. However, LukAB is phylogenetically distant to these bicomponent toxins and anti-PVL antibodies are unable to neutralize this toxin. Here, we took a systematic and rational approach to design vaccine candidates for LukAB that are attenuated, stable, and highly immunogenic, and we identified several candidates for inclusion in a multivalent toxoid vaccine.

All of the bicomponent leukotoxins, except for LukAB, are secreted as S and F subunit monomers. Cell receptor binding is initiated by S subunit followed by the binding of F and oligomerization, which leads to pore formation [6]. In contrast, LukAB is secreted as a stable dimer before engaging with the CD11b receptor molecules on the target cells following, which it hetero-oligomerizes into octomeric pore [16]. In this report, we developed a process to express LukAB from a single vector (pETDuet) to purify the protein at high yield without the use of an affinity tag. Additionally, we were able to confirm that the produced LukAB contains both S and F components and are present as dimers in solution by using reverse-phase HPLC and glutaraldehyde cross-linking. 

Badarau et al. solved the crystal structure of LukAB heterodimer in complex with a potent neutralizing antibody (ASN102) [15], as well as the octameric LukAB [11]. These studies identified 56 hydrogen bonds and four electrostatic interactions that hold the heterodimer together, as well as 34 hydrogen bonds and three electrostatic interactions that govern the formation of LukAB octamers. The authors also demonstrated that maintaining a stable dimer is critical for binding to neutralizing antibodies [15]. Badarau et al. also generated several attenuated mutants, including double mutations in LukB consisting of R23A/E and K218A or LukA D75A and D197A in interface 2 (referred to as interface 1 in [11]), which appear to interfere with octamerization. We undertook a broad screening strategy that is based on these important findings, and on the basis of LukAB sequence from USA300, to identify potential vaccine candidates that maintain LukAB structural integrity and immunogenicity, but lack toxicity. By making single and combination mutations, we were able to highlight residues in different, functional domains of the LukAB dimer that are imperative to mediating LukAB cytotoxicity. Additionally, targeting different domains on LukAB also allowed for biochemical, biophysical, and functional characterization, and delineated additional “hotspots” within LukAB dimer that act as key determinants of solubility, cytotoxicity, stability, and immunogenicity.

Of the electrostatic charges mediating protomer-protomer interactions (mediating heterodimer formation) and dimer-dimer interactions (mediating octamer formation), referred to here as interface 1 and 2, respectively, our data indicates that the residues within the salt bridges on interface 2 along with the bulky positively charged arginine and lysine residues at LukB N terminus are the key to mediating LukAB cytotoxicity. The electrostatic interactions that are part of the salt bridges within Interface 2, particularly participating LukB residues, which form a basic patch near the apical side of the cap domain, are crucial to cytotoxicity, as seen in this study. These residues have been previously shown to be important for LukAB octamerization and are known to be fully conserved between the LukAB sequence variants [11]. In contrast, the salt bridge residues mutated within Interface 1 did not contribute to cytotoxicity as much. In our observation, substituting charged residues (K12/K19 and R23) or swapping of the first 30 residues of LukB N terminus with those of HlgB exhibited the highest impact on cytotoxic function. A previous report by DuMont et al. showed that the deletion of the first 33 residues of LukA does not affect copurification with LukB or heterodimer formation, but increases pore formation, thereby moderately increasing cytotoxicity [12]. Our data indicates that, unlike LukA, the N terminus of LukB is indispensable for both heterodimer formation and cytotoxicity. While the N-terminal 30 residues of HlgB transplanted to LukB allow for heterodimer formation, it does not restore cytotoxicity, which is likely because of the loss of R23 that is critical for the salt bridge that is involved in dimer-dimer interaction. In contrast, the complete removal of those 30 residues resulted in the failure to express LukB. These observations are reminiscent of the functional properties of the amino latch that are found at the N terminus of α-toxin, where the residues control hemolytic activity and remain central to protomer-protomer interactions [30]. Interestingly, the N terminal residues of LukB are unique to LukAB and they are not conserved among other S and F components or α-toxin alluding to a possibility that LukB N terminus may be functionally unique with putative roles in oligomerization and toxicity, requiring further investigation. 

Mutations that were made to disrupt the buried hydrophobic pocket found within Interface 1, particularly L61, was found to be crucial to LukAB thermostability and attenuating toxicity; however, drastically hampering immunogenicity. As this residue is not surface exposed, the pocket near L61 is not an antigenic target, but it lowers immunogenicity, which is likely due to altered oligomeric status. The structures of LukAB and α-toxin show that L61 is nestled between β-strands that are part of the β-sandwich within the cap domain and the introduction of bulky mutations perturbs the hydrophobicity within this pocket affecting crucial protomer-protomer interactions that are sacred to stability, thereby reducing its ability to generate neutralizing antibodies. 

In our efforts, we have been successful in identifying a strong vaccine candidate in LukA_D39A_LukB_R23E_ that satisfied biochemical and biophysical characterization, in addition to showing complete attenuation in PMNs. It is interesting that the second-best candidate, LukA_wt_LukB_HlgB_ mutant, which exhibited the complete attenuation in PMN lytic activity, retained some toxicity, albeit at high concentrations in the THP-1 cells, suggesting that the substituted HlgB residues may be responsible for the relapsed cytotoxic effects. Interestingly, both of these mutants were able to compete with WT LukAB toxicity at a low molar ratio of below 1, which indicated that the competition cannot be entirely due to receptor binding by the mutant. The reversal of LukAB toxicity by these mutants may relate to the formation of defective mixed oligomeric structures, an observation that warrants further investigation. Moreover, with this study, we can show that the identified vaccine candidate generates antibodies that can reverse the toxicity and show protection in PMNs, as well THP-1 monocytes.

Previous studies showed the important role of LukAB in cell specific lysis of monocytes [14] and dendritic cells (DC) [31], as well as its role in macrophage dysfunction [17]. However, these activities are also shared by some of the other pore-forming toxins. Most of the strains that were tested in our study showed clear synergism between polyclonal antibodies against LukAB and other pore-forming toxins. Cocktail polyclonal: αHla, LukS-PV, and LukF-PV (generated against three toxoid proteins) alone were unable to neutralize the toxicity of culture supernatants from most of the virulent strains in PMN lysis study. However, when this cocktail polyclonal was mixed with anti-LukAB polyclonal antibodies, 100% neutralization of culture supernatants was achieved, indicating the importance of neutralizing all the pore-forming toxins. 

In summary, in this study, we have developed at least two vaccine candidates for LukAB, which is an important virulence factor of *S. aureus*. Our findings indicate that a multivalent approach targeting the related leukotoxins PVL, LukED, HlgAB, HlgCB, and the divergent LukAB, as well as the single component Hla is critical for protection against cytolytic activity of the most prevalent *S. aureus* strains. These data strongly support the development of a multivalent toxoid vaccine for *S. aureus,* which covers all major pore-forming toxins.

## 4. Materials and Methods

### 4.1. Generation of LukAB Wild-Type (WT) and Mutants in pET Duet and pET24a (+)

General methods that are used for bacterial culture have been described previously in detail (18,19). In this study, we optimized LukAB expression while using two different vectors: pET Duet-1 (Novagen), where LukA was cloned into the multiple cloning site 1 (MCS1) while using *Nco*I-*Hind*III, and LukB was cloned into MCS2 using *Nde*I-*Xho*I restriction sites within the same vector. In another system, LukA and LukB were expressed while using two different pET-24a(+) plasmids within the same *E. coli* cell. We compared LukAB WT expression, yield, and toxicity from these two systems. All of the mutants were expressed while using the latter system, where LukA and LukB were expressed using separate plasmids within the same *E. coli*. Towards this end, LukA (WT or mutants) was cloned into pET-24a(+) with a Kanamycin resistant marker within *Nde*I-*Xho*I sites. Similarly, LukB (WT/mutants) was inserted within *Nde*I-*Xho*I sites, but we replaced the inherent Kanamycin resistant cassette in pET-24a(+) with Ampicillin. The origin of replication of pET-24a(+) was replaced by p15a resulting in a recombinant pET24a(+)Amp^R^p15a LukB vector to increase the plasmid compatibility. This LukB plasmid (WT/mutant) was transformed into BL21(DE3) (NEB) containing pET24a(+) LukA plasmid (WT/mutant) and the colonies were selected on LB plates with 50 ug/mL of Kanamycin (Kan_50_) and 100 ug/mL of Ampicillin (Amp_100_). The genes for LukAB (USA300_TCH1516) WT and mutants were codon optimized prior to transformation by GenScript^®^.

### 4.2. Growth Media and Bacterial Strains

Overnight cultures of *E. coli* that were grown at 37 °C in LB Kan_50_Amp_100_ were expanded to 0.5 L in a shaking incubator (225 rpm), until they reached an OD OD_600_ of 0.5. The cultures were immediately chilled on ice for 10 min. with periodic shaking and then induced with 0.3 mM IPTG (Sigma, St.Louis, MO, USA) in a shaking incubator (225 rpm) overnight at 25 °C. The following day, the cells were harvested by centrifugation (14,000× *g*) and frozen at −80 °C. To lyse the cells, the pellet was resuspended in 3 mL of cell lysis buffer (20 mM Tris pH 8.0, 50 mM NaCl, 1 mM EDTA, 0.1% Triton X-100) per gram of cell paste and Hen egg lysozyme (Sigma, St. Louis, MO, USA) at 1 mg/mL final concentration prior to incubation at 37 °C for 30 min. The partially lysed cells were then cooled in a wet/dry ice ethanol bath, followed by sonication while using a microtip (10 × 10 s bursts with cooling between bursts, output 5, 50% duty). Lysis was confirmed by measuring the reduction of OD_600_ absorbance. Post lysis, the NaCl concentration was adjusted to 0.5 M and nucleic acid precipitation was carried out by the dropwise addition of 0.3–0.5% polyethyleneimine (PEI) while maintaining constant mixing. The PEI pellet was separated by centrifugation at 12,000 rpm in a Sorvall SS34 rotor and the supernatant containing the toxoid was subjected to ammonium sulphate (AmS0_4_) precipitation. Towards this end, 0.472 g/mL of AmS0_4_ powder was added to the PEI supernatant and then placed on a rotating mixer for 20 min. at room temperature. The resulting pellet that was obtained by centrifugation at 12,000× *g* for 30 min. at 4 °C was frozen at −80 °C until purification. 

The AmS0_4_ pellet was resuspended and buffer exchanged into 20 mM NaPi pH 6.5, 25 mM NaCl, 5% glycerol using a GE Healthcare PD10 desalting column. The mixture was then clarified by filtration using 0.8/0.2 µm Supor^®^ low protein binding syringe filter (Pall Life Sciences, Port Washington, NY, USA). The toxoid from the resulting solution was purified while using a two-column purification approach. The first purification was carried over a 10 mL Poros 50 HS column using a 40-column volume (C.V.) gradient from 25 to 1000 mM NaCl in the phosphate buffer. The peak fractions were analyzed by standard SDS-PAGE analysis and accordingly pooled together for dialysis into 20 mM NaPi pH 6.8, 50 mM NaCl, 5% glycerol, the equilibration buffer for the second column—a 10 mL ceramic Hydroxyapatite (HTP) (BioRad, Hercules, CA, USA; Type 1 40 µm). The toxoid was eluted using a 40 C.V. gradient from 50 to 1000 mM NaCl in the phosphate buffer. The appropriate fractions were pooled together upon SDS-PAGE evaluation and then dialyzed into the final storage buffer (20 mM NaPi pH 7.4, 150 mM NaCl, 5% glycerol). All purified LukAB proteins (MW = ~72 kDa) were concentrated while using Amicon 3K MWCO Ultra 15, filtered through 0.8/0.2 µm low protein binding membrane, and stored at −80 °C prior to use.

### 4.3. Growth Media and Bacterial Strains

Table S2 lists the bacterial strains that were used in this study. The SA strains were grown in brain heart infusion broth/agar (BHI) and tryptic soy broth/agar (TSB) media at 37 °C, whichever appropriate. The overnight bacterial culture supernatants were normalized based on culture OD_600_ absorbance. The next day, culture supernatants were filtered through 0.2 µm filter to sterilize the supernatants. Sterility was confirmed by culturing 100 μL of the filtered supernatants on BHI or TSA agar plates overnight. 

### 4.4. Cell Culture Maintenance and Induction

The HL-60 cells (ATCC, Manassas, VA, USA) were cultured in RPMI 1640 (Gibco, Gaithersburg, MD, USA) supplemented with 16.4% heat inactivated fetal bovine serum (FBS), 4 mM L-glutamine, 82 U/mL each of penicillin, and streptomycin. Cells were passaged twice a week at a concentration of 6–8 × 10^5^ cells/mL. 1 × 10^7^ cells were seeded in 30 mL and grown in culture media with 1.5% dimethyl sulfoxide (DMSO) for seven days to differentiate cells into neutrophils. CD11b expression using flow cytometry confirmed induction.

### 4.5. PMN-based Cytotoxicity Assay

The induced HL-60 cells were harvested by centrifugation at 420× *g* (Sorvall RT6000B rotor, ThermoFisher Scientific, Waltham, MA, USA) for 10 min. at 20 °C. Cells were washed and resuspended with phenol red-free RPMI 1640 (Gibco, Gaithersburg, MD, USA) supplemented with 2% FBS to a final concentration of 5 × 10^6^ cells/mL. LukAB mutants (proteins) were serially diluted two-fold across 96-well plates (50 µL/well) and 100 µL of 5 × 10^6^ cells/mL were added to each well. The plates were incubated at 37 °C, 5% CO_2_, 95% humidity for 3 h. After 3 h, either XTT (Cell Signaling Technology) or CellTiter Glo (Promega) reagent was used to determine the cell viability and cytotoxicity [21,32]. When using XTT for readout, 50 µL of the activated XTT (50 μL electron coupling per 5 mL XTT) reagent was added to each well, and plate was returned to 37 °C, 5% CO_2_, 95% humidity for 16–18 h. After incubation, the cells were pelleted by centrifugation at 3500 rpm 3565.9× *g* (Sorvall RT6000B rotor, ThermoFisher Scientific, Waltham, MA, USA) for 3 min. The supernatants were transferred to 96-well ELISA plates and absorbance was read at 470 nm while using Spectramax 190 plate reader (Molecular Devices, San Jose, CA, USA) and Softmax 5.4.5 software (Molecular Devices, Waltham, MA, USA). When using CellTiter Glo, 50 μL of the reconstituted CellTiter Glo reagent was added to each well. The plate was shaken on an orbital shaker for 10–15 min., followed by measurement of luminescence (emission at 560 nm) using Cytation 5 imaging reader (Biotek, Winooski, VT, USA) and Gen5 2.09 software to determine cell viability. For the kinetic cytotoxicity studies, the replicates were incubated for 6 and 19 h, in addition to a 3-h incubation period. After incubation, the CellTiter Glo reagent was used to determine the cell viability.

### 4.6. Rabbit Polyclonal Antibody Generation

Rabbit polyclonal antibody generation for anti-Hla_DM_, LukS_mut1_, LukF_mut9_, and LukA_D39A_B_R23E_ toxoid as immunogens were generated by Genscript^®^ (Piscataway, NJ, USA) using >98% pure proteins as immunogens. Briefly, four New Zealand white rabbits were immunized per toxoid on day 0, 14, and 21 with 0.2 mg protein per rabbit, along with Freund’s Incomplete Adjuvant subcutaneously. The test bleeds and production bleeds were collected on day 21 and day 42. Hyperimmune sera were individually characterized for ELISA titer and TNA titers before pooling them together. The pooled serum was purified by Protein A affinity chromatography into total IgG and labeled, as follows: anti-Hla (IBT Cat: 1940-01 Rb pAb), anti-LukS- (IBT Cat: 1941-01 Rb pAb), anti-LukF-PV(IBT Cat: 1942-01 Rb pAb), and anti-LukAB (IBT Cat: 1944-02 Rb pAb). Full quality control (QC) were performed before use.

### 4.7. Toxin Neutralization Assay (TNA) in PMNs

The serum samples were serially diluted two-fold in RPMI across 96-well plates (25 µL/well). Twenty-five microliters of 2.5 nM LukAB toxin was added to each well. 100 µL of induced cells were prepared, as described above, at 5 × 10^6^ cells/mL were added to each well. The plates were incubated at 37 °C, 5% CO_2_, 95% humidity for 3 h, followed by XTT or CellTiter Glo readout to determine the cell viability and neutralization [21,32]. 

### 4.8. Reverse-TNA in PMNs

Select LukAB mutants were serially diluted from a starting concentration of 12 nM in RPMI semi-log across 96-well plates (25 µL/well). Polyclonal αLukAB at 100 µg/mL or RPMI was added to each well (12.5 µL/well), followed by incubation at RT for 10 min. Following incubation, 12.5 µL of 5 nM LukAB WT and 100 µL of 5 × 10^6^ induced HL-60 cells/mL were added to each well. The plates were then incubated for 3 h at 37 °C, 5% CO_2_, 95% humidity, followed by CellTiter Glo readout determine cell viability and neutralization. 

### 4.9. Differential Scanning Fluorimetry (DSF)

The proteins in the storage buffer (20 mM NaPi pH 7.4, 150 mM NaCl, 5% glycerol) were mixed with 2X SYPRO orange dye (Invitrogen, Carlsbad, CA, USA) in a 96-well hard shell plate with clear bottom (BIO-RAD, Hercules, CA, USA) and then placed into a thermal cycler, wherein the temperature scan rate was fixed at 0.5 °C/min over a range of 30–99 °C (21). The fluorescence intensities were plotted against temperature to get a sigmoidal curve and the melting temperatures (Tm) were calculated while using the first derivative. BSA (Pierce) was used as a control, which recorded a melting temperature of 66 ± 0 °C in 1 × PBS pH 7.4. 

### 4.10. Cross-Linking with Glutaraldehyde

LukAB_WT_ and mutants at 50 ug/mL concentration were incubated with 0.25% glutaraldehyde (Sigma, St. Louis, MO, USA) for 2 min. at 37 °C in a final volume of 100 μL in 20 mM HEPES pH 7.5, 50 mM NaCl. The reaction was stopped by adding 10 μL of 1M Tris, pH 8.0 and 4 × LDS sample buffer, followed by SDS-PAGE and Western blot analysis while using αLukAB and rabbit pAb αLukB-specific (IBT Cat: 0313-001). αLukS-PV and αLukF-PV were used as the negative controls. 

### 4.11. Reverse-Phase HPLC

LukAB (WT/Mutant; 100 μg) was injected into an AdvanceBio RP-mAb diphenyl column (Agilent 795975-944, Santa Clara, CA, USA, 4.6 × 100 mm, 3.5 micron) in a 1260 Infinity Quaternary instrument. A 30–90% gradient method was used consisting of 100% acetonitrile in Line A and 0.1% TFA (*v*/*v*) in Line B with a flow rate of 1.00 mL/min over 60 min. and a column temperature of 27 °C.

### 4.12. Animals and Immunizations

Female ICR (CD-1) mice, six weeks of age, were purchased from Envigo (US). The mice were maintained under pathogen-free conditions and fed laboratory chow and water ad libitum. All mouse work was conducted in accordance with protocols that were approved by institutional animal care and use committees (IACUC) of Integrated BioTherapeutics, where mouse studies were performed (approval date 28 February 2017; approval code: D17-00974). The mice were intramuscularly immunized (IM) three times two weeks apart with 10 µg of LukAB mutant in 50 µg of Al(OH)_3_. For serological analysis, the mice were test bled via retro-orbital (RO) route prior to and 10 days after the third and final immunization.

### 4.13. Enzyme-Linked Immunosorbent Assay (ELISA) for Determination of Serum Titers

Blood samples from mice were centrifuged in serum separator tubes and the serum samples were stored at −80 °C until further use in ELISA. Briefly, 96-well plates were coated with 300 ng/well of LukAB WT overnight at 4 °C. The plates were blocked with Starting Block (SB) (Thermo Scientific, Waltham, MA, USA) for one hour at room temperature (RT). Serum samples were diluted in a semi-log manner starting from 1:100 to 1:316,228 in a 96-well plate, using starting block buffer as the diluent. The plates were washed three times and sample dilutions were applied in 100 µL volume/well. The plates were incubated for 1h at RT and washed three times before applying the conjugate, goat anti-mouse IgG (H+L)- Horseradish Peroxidase (HRP) in SB. Plates were incubated for 1 h at RT, washed, as described above, and incubated with TMB (3,3′,5,5′-tetramethylbenzidine) for 30 min. to detect HRP activity. Optical density at 650 nm was measured while using a Versamax™ plate reader (Molecular Devices, Waltham, CA, USA). Data analysis for full dilution curves was performed using Softmax program and graphed in GraphPad Prism.

### 4.14. Cytotoxicity Assay in Human Monocytes and THP-1 Cells

Purified human monocytes (CD14^+^) up to 95.98% purity were purchased from BioIVT (San Carlos, CA, USA) and kept frozen in LiN_2_ until use. Acute monocytic leukemia THP-1 cells were purchased from ATCC (ATCC^®^ TIB-202™) and were cultured at 4E5 cells/mL every 2–3 days at 37 °C + 5% CO_2_, as recommended in RPMI-1640, supplemented with 0.05 mM 2-mercaptoethanol and 10% FBS. To differentiate the THP-1 cells, 100 nM of Phorbol 12-myristate 13-acetate (PMA; Sigma, St. Louis, MO, USA) was added to THP-1 media and seeded at 4E5 cells/mL. The cells were allowed to differentiate for three days and analyzed for extent of cell adherence and surface marker for differentiation (i.e., CD11b) by flow cytometry. After three days, the media was replaced with non-PMA containing medium and allowed to rest for five days before use, feeding the cells with fresh THP-1 medium every two days. 

To evaluate the cytotoxicity effects of purified LukAB (WT/mutants) or other toxins, such as α-toxin, LukSF, HlgAB, and HlgCB on differentiated THP-1 cells, the adherent cells were harvested by treatment with 1 × PBS + 0.05 mM EDTA at 37 °C for ~5 min. and immediately centrifuged at 420× *g* (Sorvall RT6000B rotor) for 10 min. The cells were then washed twice in 1% (*w*/*v*) RPMI+Cas (Bacto BD, Franklin Lakes, NJ, USA) and pelleted by centrifugation. Proteins (12.5 µL) were serially diluted two-fold or in semi-log fashion, as indicated in 1% RPMI+ Cas and incubated with 75 µL cells (seeded at 1E5 cells/well in a 96-well plate) in a final volume of 125 µL for 1 h at 37 °C, 5% CO_2_, 95% humidity, followed by CellTiter Glo readout to determine the cell viability and % lysis. For experiments with supernatants from culture filtrates, the supernatants (as listed in Table S2) were normalized to OD_600_ = 6 prior to serial dilution in RPMI+Cas. The incubation times were increased to 4 h at 37 °C, 5% CO_2_, 95% humidity prior to CTG treatment and readout. For conditions with a polyclonal antibody, 12.5 µL of single or each antibody was added to the well, followed by incubation with purified protein or supernatant for 10 min. at RT prior to the addition of cells. Cells incubated with a final concentration of 0.2 or 0.4% Triton-X-100 in 1% RPMI+ Cas was used as the control for complete lysis. 

### 4.15. Flow Cytometry Analysis

HL60, DMSO-treated HL-60, THP-1, and PMA-treated THP-1 cells were seeded at 1.5E5 cells/well on a clear 96-well plate. The cells were washed with 200 µL/well of PBS without Ca^2+^ and Mg^2+^, supplemented with 2% FBS (FACS buffer). For HL60 cells, CD11b-FITC (BDPharmingen; Cat: 562,793 Clone: ICRF44) was added to each well at 1:40 dilution (50 µL) in FACS buffer and incubated at RT, being covered from light, for 15 min. For THP-1 cells, a combination of CD11b and CD14-APC/Cy7 stains (Cat: 301,820 Clone: M5E2) were used to monitor CD11b upregulation in addition to macrophage differentiation. The cells were washed twice with FACS buffer at 1400 rpm for 5 min. each and LIVE/DEAD Fixable Near-IR Dead Cell stain (ThermoFisher Scientific, Waltham, MA, USA) was added to each well at a dilution of 1:500 (50 µL) and incubated on ice, covered from light, for 15 min. The cells were washed again and resuspended in 200 µL FACS buffer. Fluorescence measurements were acquired using either Guava flow cytometer (EMD Millipore, Burlington, MA, USA) or they were acquired at a Symphony A3 (BD Biosciences, Franklin Lakes, NJ USA). Data was analyzed with FlowJo software V10. Induction was considered to be successful if CD11b expression in viable cells was found to be ~70% or higher. 

## 5. Patents

An international patent application (WO2018232014A1) has been filed covering the composition of matter and method of used disclosed in this report.

## Figures and Tables

**Table 1:** Characteristics of the LukAB mutants, including cytotoxicity, production yield, and immunogenicity.

LukA	LukB	Location	Tox. EC_50_ (nM)	Relative Attenuation (Mutant/WT)	Expression (mg/L)	ELISA EC_50_	TNA NT_50_	EC_50_ Mut/WT	NT_50_ Mut/WT
WT	WT	WT	0.03	1.00	20.574	17800	2260	1	1
L61R	WT	Interface1	>1000	>36,752	4.6	9000	1290	0.51	0.57
L61N	WT	Interface1	0.72	26.36	8.82	ND	ND		
L61Q	WT	Interface1	0.11	4.06	8.98	ND	ND		
WT	D49A	Interface1	0.11	4.04	3.765	ND	ND		
WT	D49K	Interface1	0.01	0.35	9.6	ND	ND		
WT	R23A	Interface2	>1000	>36,752	8.616	1320	597.5	0.07	0.26
WT	R23E	Interface2	>1000	>36,752	3.384	16,500	1540	0.93	0.68
WT	K58E	Interface2	>1000	>36,752	7.056	ND	ND		
WT	K58A	Interface2	>1000	>36,752	4.384	14,700	5900	0.83	2.61
WT	E112A	Interface2	0.12	4.35	40.72	5070	3010	0.28	1.33
D39A	WT	Interface2	0.04	1.36	9.648	16,500	3170	0.93	1.40
D39A	R23E	Interface2	>1000	>36,752	23.24	14,000	2640	0.79	1.17
D39R	R23E	Interface2	>1000	>36,752	21.64	682	334	0.04	0.15
D39R	K218A	Interface2	0.95	35.01	20.84	ND	ND		
D39A	E112A	Interface2	0.06	2.04	29.6	4770	4060	0.27	1.80
K133A	K218A	Interface2	0.05	1.89	42.56	2320	1570	0.13	0.69
D39R	E112A	Interface2	0.00	0.13	55.2	1800	765	0.10	0.34
L61R	R23E	Interface1&2	>1000	>36,752	31.6	1560	219	0.09	0.10
L61R	R23A	Interface1&2	>1000	>36,752	30.2	863	164.5	0.05	0.07
L61R	K218A	Interface1&2	>1000	>36,752	21.28	2640	162	0.15	0.07
D197K	R23A	Interface1&2	>1000	>36,752	34.04	1610	764	0.09	0.34
D39A	K218A	Interface1&2	0.00	0.02	50.4	2740	748	0.15	0.33
L61R	E112A	Interface1&2	99.00	3638	39.8	12,700	2100	0.71	0.93
WT	HlgB	Interface 2 (N Term)	>1000	>36,752	28	15,600	2110	0.88	0.93
WT	K12/K19/R23A	Interface 2 (N Term)	15.61	573.7	17.72	3120	1050	0.18	0.46
L61R	K12/K19/R23A	Interface1 & 2(N Term)	>1000	>36,752	35.8	2310	613	0.13	0.27
L61R	HlgB	Interface1 & 2(N Term)	>1000	>36,752	14.33	22,900	611	1.29	0.27
D39A	K12/K19/R23A	Interface2+(N Term)	>1000	>36,752	23.52	965	1030	0.05	0.46
D39R	K12/K19/R23A	Interface2+(N Term)	>1000	>36,752	26.8	942	770	0.05	0.34
D197A	WT	MBC	0.16	5.94	2.96	ND	ND		
WT	E197A	MBC	0.12	4.51	ND	ND	ND		
WT	H180A	MBC	0.00	0.11	4.18	ND	ND		
WT	R203A	MBC	0.00	0.10	27.16	4840	1100	0.27	0.49
D197K	WT	MBC	0.00	0.06	14.74	ND	ND		
L61R	R203A	Interface1& MBC	>1000	>36,752	27.4	ND	ND		
D39R	R23E/R203A	Interface1, 2 & MBC	>1000	>36,752	15.88	ND	ND		
WT	125-133_1G	Pore	33.16	1218.7	did not elute	47,100	2570	2.65	1.14
WT	127-133_APGP	Pore	0.26	9.38	19.4	ND	ND		
WT	127-33_SNGLS	Pore	0.00	0.01	20.52	5000	3380	0.28	1.50

* MBC: Membrane Binding Cleft; ND: Not determined.

## Supplementary Materials

The following are available online at https://www.mdpi.com/2072-6651/11/6/339/s1, Table S1: Comparison of expression using pET Duet versus pET24a (+) dual plasmid system, Table S2: Bacterial Strains, Figure S1: Key residue mutations within LukAB, Figure S2: Expression of CD11b and/or CD14 on (**A**) PMN and (**B**) THP-1 cells.
