# Supplementary material for: Rational Design of Toxoid Vaccine Candidates for Staphylococcus aureus Leukocidin AB (LukAB)
Source: Toxins (Basel). 2019 Jun 14;11(6):339. doi: 10.3390/toxins11060339 (PMC6628420; doi:10.3390/toxins11060339)
Supplement: Supplementary file 1 [file toxins-11-00339-s001.pdf]

Supplementary Materials: Rational Design of Toxoid Vaccine Candidates for *Staphylococcus aureus* Leukocidin AB (LukAB)

Shweta Kailasan, Thomas Kort, Ipsita Mukherjee, Grant C. Liao, Tulasikumari Kanipakala, Nils Williston, Nader Ganjbaksh, Arundhathi Venkatasubramaniam, Frederick W. Holtsberg, Hatice Karauzum, Rajan P. Adhikari and M. Javad Aman

Table S1. Comparison of expression using pET Duet versus pET24a (+) dual plasmid system.

Vector	IPTG Concentration (mM)	Growth/ Induction Temperature (°C)	Total Yield (mg/L)	EC50 from PMN-Based Toxicity Assay (nM)
pET Duet	0.3	37/25	19.84	0.1
pET24a(+)	0.3	37/25	20.5	0.09

Abbreviations: IPTG (Isopropyl β-D-1-thiogalactopyranoside); PMN (Polymononuclear neutrophils)

Table S2. Bacterial Strains.

Strain	Genotype/Description	Source
JE2	USA300 WT from Nebraska mutant library	NARSA repository
MRSA252, MN8, NCTC 8325, MRSACOL, USA200, USA100, MNHOCH	Standard SA strain	NARSA repository
MRSA ST-80	Standard SA strain	From Dr. Jean Lee, BWH, Harvard University
USA300 (NRS384)<br>USA1000, SF8300 and isogenic LukAB mutant	Community MRSA strain<br>Community MRSA strain and it's isogenic LukAB mutant	NARSA repository<br>From Dr. Binh Diep, UCSF

Abbreviations: SA (Staphylococcus aureus); MRSA (multi-drug resistant SA)

LukA$_{wt}$

```
MKNKKRVLIA SSLSCAILLL SAATTQANSA
        :* *********: *::*******

HKDSQDQNKK EHVDKSQQKD KRNVTNKDKN     33
 ********* :**** ***. * *  :* :*

STAPDDIGKN GKITKRTETV YDEKTNILQN     63
* . ::* **  ********* **:*******

LQFDFIDDPT YDKNVLLVKK QGSIHSNLKF     93
***:** *** ****:***** ** *******

ESHKEEKNSN WLKYPSEYHV DFQVKRNRKT    118
:*********  ****** ** **:**** **

EILDQLPKNK ISTAKVDSTF SYSSGGKFDS    153
***:** *** *** **:.** ****.*****

TKGIGRTSSN SYSKTISYNQ QNYDTIASGK    183
 ********:. ********** :**:*: .*

NNNWHVHWSV IANDLKYGGE VKNRNDELLF    213
*****:***:* *****:*** ***:******

YRNTRIATVE NPELSFASKY RYPALVRSGF    243
***:****** ********** *  *** *.*

NPEFLTYLSN EKSNEKTQFE VTYTRNQDIL    273
* ******** ******   ** *****:*:**

KNRPGIHYAP PILEKNKDGQ RLIVTYEVDW    303
*** *:**.*  * ****:** **** *****

KNKTVKVVDK YSDDNKPYKE G             324
:*:* *.*** ::*::  ::  .
```

LukB$_{wt}$

```
MIKQLYKNIT ICTLALSTTF TVLPATSFAK      1
                                *

INSEIKQVSE KNLDGDTKMY TRTATTSDSQ     31
*:**** :** ***:.****  ***** ***:

KNITQSLQFN FLTEPNYDKE TVFIKAKGTI     61
*****:** *:*: ****  **:** ****

GSGLRILDPN GYWNSTLRWP GSYSVSIQNV     91
******   * ******* *  * *****:**

DDNNNTNVTD FAPKNQDESR EVKYTYGYKT    121
::* :*:**: *  ***.***  ***** ***

GGDFSINRGG LTGNITKESN YSETISYQQP    151
.********. ***** **** * * ******

SYRTLLDQST SHKGVGWKVE AHLINNMGHD    181
********** *:**:***** *:*******

HTRQLTNDSD NRTKSEIFSL TRNGNLWAKD    211
********** *****:**** **. .** **

NFTPKDKMPV TVSEGFNPEF LAVMSHDKKD    241
*** *:*: * .*** .**** *::*******

KGKSQFVVHY KRSMDEFKID WNRHGFWGYW    271
:***:***** ** :****:* **********

SGENHVDKKE EKLSALYEVD WKTHNVKFVK    301
*******:** ***** ***  :*** :**:*

VLNDNEKK                            331
*** ****
```

Figure S1. Key residue mutations within LukAB. LukAWT and LukBWT sequences from USA300 strain are shown along with specific mutated residues highlighted in yellow. For LukB, residues crossed out indicate deletion mutations and those colored in blue were substituted with the corresponding residues in HlgB (AEGKITPVSVKKVDDKVTLYKTTATADSD). The leader or signal sequence is shown in bold & italics. Additionally, conservation of residues within LukAWT and LukBWT variants derived from different strains for which annotated genome data is available is shown below the residues as calculated from a multiple sequence alignment using ClustalOmega (Ref). The symbols indicate conservation of: a single residue (*), between groups of strongly similar properties - scoring > 0.5 in the Gonnet PAM 250 matrix (:), and between groups of weakly similar properties - scoring ≤ 0.5 in the Gonnet PAM 250 matrix (.).

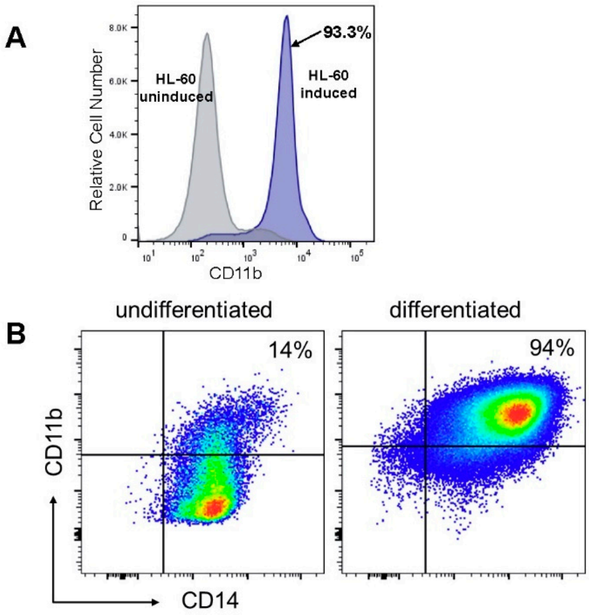

Figure S2. Expression of CD11b and/or CD14 on (**A**) PMN and (**B**) THP-1 cells.
